# Enhancing the Electrochemical Performance of High Voltage LiNi_0.5_Mn_1.5_O_4_ Cathode Materials by Surface Modification with Li_1.3_Al_0.3_Ti_1.7_(PO_4_)_3_/C

**DOI:** 10.3390/nano13040628

**Published:** 2023-02-05

**Authors:** Tingting Yang, Chi-Te Chin, Ching-Hsiang Cheng, Jinsheng Zhao

**Affiliations:** 1 School of Automotive Engineering, Wuhan University of Technology, Wuhan 430070, China; 2 Hubei Key Laboratory of Advanced Technology for Automotive Components, Wuhan University of Technology, Wuhan 430070, China; 3 Hubei Research Center for New Energy & Intelligent Connected Vehicle, Wuhan University of Technology, Wuhan 430070, China; 4 College of Chemistry and Chemical Engineering, Liaocheng University, Liaocheng 252059, China

**Keywords:** solvent recrystallization, lithium iodide, LATP/C composite, LiNi_0.5_Mn_1.5_O_4_ cathode, low temperature

## Abstract

A novel method for surface modification of LiNi_0.5_Mn_1.5_O_4_ (LNMO) was proposed, in which a hybrid layer combined by Li_1.3_Al_0.3_Ti_1.7_(PO_4_)_3_ (LATP) and carbon (C) composite on LNMO material were connected by lithium iodide. Structure and morphology analyses illustrated that a higher contact area of active substances was achieved by the LATP/C composite layer without changing the original crystal structure of LNMO. XPS analysis proved that I^−^ promoted the reduction of trace Mn^4+^, resulting in a higher ion conductivity. Galvanostatic charge–discharge tests exhibited the capacity of the LNMO with 5% LATP/C improved with 35.83% at 25 °C and 95.77% at 50 °C, respectively, compared with the bare after 100 cycles, implying the modification of high-temperature deterioration. EIS results demonstrated that one order of magnitude of improvement of the lithium-ion diffusion coefficient of LATP/C-LNMO was achieved (3.04 × 10^−11^ S cm^−1^). In conclusion, the effective low-temperature modification strategy improved the ionic and electronic conductivities of the cathode and suppressed the side reactions of high-temperature treatment.

## 1. Introduction

Due to the continuous upgrading of various types of consumer electronics, the demand for lithium-ion batteries of small size, light weight, and high energy density has been increasing [1,2]. Increasing the operating voltage is a significant strategy to improve the battery’s energy density, so exploring high-voltage cathode materials has attracted attention, especially in the application of electric vehicles. Among various cathode materials, LNMO ($Fd\bar{3}m$) [3,4] has advantages such as a high operating voltage (4.7 V and 4.0 V vs. Li/Li^+^) [5], relatively high theoretical specific energy (650 wh kg^−1^), good rate performance, abundant resources, and low price (it is cobalt free). The voltage platform of 4.7 V is attributed to Ni^2+^ → Ni^4+^ redox reactions, while the platform around 4.0 V mainly depends on Mn^3+^ → Mn^4+^ redox reactions [6,7,8]. At the same time, LNMO has a three-dimensional lithium-ion transfer path and a high electron conductivity (10^−5^~10^−7^ S/cm), which makes it a promising candidate for the high voltage cathode of a lithium-ion battery.

However, the rapid decay of the capacity of LNMO caused by various problems limits its commercialization. In particular, the HF and other substances generated by the decomposition of liquid electrolytes at high temperatures and high voltage corrode the surface of the cathode material and the LiF and MnF_2_ substances that prevent the interfacial charge and ion transfer, which finally affects the cycle stability of LNMO [9,10,11]. The direct contact between the cathode material and electrolyte will dissolve the Mn element on the surface, and the high-valent Mn^4+^ will be reduced to Mn^3+^ or Mn^2+^, while the disproportionation reaction of Mn^3+^ will promote the further dissolution of Mn, resulting in structural collapse and large volume change [11,12]. However, only a moderate increase in Mn^3+^ in LNMO can enhance the conductivity of ions and electrons, benefiting the enhancement of rate performance mm [13,14]. In high-voltage cycles, the self-discharge behavior of LNMO [15] and the presence of water impurities [5] are all factors that contribute to the production of HF in the electrolyte, which further promotes the dissolution of surface metal. In addition, LNMO high-voltage materials react in solid–liquid two-phase reactions in the voltage range of Ni^3+^ →Ni^4+^, resulting in grain boundary movement and the generation of intracrystalline stresses [16]. The above factors are not favorable to the LNMO cathode material’s ability to maintain structural stability during the cycle, which ultimately leads to poor cycle stability performance and magnification performance. Thus, it is necessary to improve the electrode–electrolyte interface to reduce the structural changes of the LNMO electrode to make it more suitable for high-voltage lithium-ion batteries.

Surface coating is an effective method used to reduce the occurrence of interfacial side reactions and improve the electrochemical properties of cathode materials. Metal oxides (SiO_2_ [13,17], Al_2_O_3_ [18,19], ZrO_2_ [20], etc.), phosphates (Li_3_PO_4_ [21], LiCoPO_4_ [22], Li(Co_0.5_Ni_0.5_)PO_4_ [23], etc.), conductive polymers [24] and other coatings can reduce the direct contact between the cathode material and the electrolyte to a certain extent, effectively enhancing the structural stability and acid corrosion resistance of LNMO. Carbon is an excellent conductor of electrons and is generally used as a conductive agent for cathode materials [25]. A variety of organic polymers (polypyrrole (PPY) [24,26], polyacrylonitrile (PAN) [27], glucose [28], polymethyl methacrylate—polyethene glycol (PMMA-PEG) [29], polymer polyaniline [30], etc.) are used as carbon sources, forming carbon nano-coated layers on the surface of LNMO after high-temperature carbonization. This has been shown to improve the conductivity of materials and provide stable chemical and electrochemical reaction interfaces [31,32]. However, a too-thick carbon coating could block the transmission channels of lithium ions, reducing the specific energy of the material [33]. At the same time, carbon coating generally requires a high-temperature treatment process in an inert gas, especially for organic carbon sources (glucose, acetylene gas [34], etc.), but higher temperatures pose a risk of side reactions, such as a reduction in metal oxide in the cathode material, which is caused by carbon [35,36]. Among the various coating materials, solid electrolytes (oxide solid electrolytes LATP and LLZO, etc.) are more promising coating materials thanks to their high lithium-ion conductivity [37,38] (10^−4^~10^−3^ S cm^−1^) and wide operating range, so more and more research has begun to focus on the use of suitable solid electrolytes to build a stable and compatible interface for LNMO-based batteries [39,40,41]. In this study, NASION-type LATP solid-state electrolytes were selected as the primary part of the composite coating layer, improving electrochemical properties by stabilizing the structure. LATP has excellent ionic conductivity in both crystalline and amorphous states while exhibiting excellent structural stability under high pressure. In previous studies, LATP was used as a coating layer for cathode materials (NCM [42,43,44], LCO [45], LNMO [39,46,47], etc.) mainly employing the use of high-temperature treatment (≥400 °C) processes to obtain a uniform and stable surface structure [41,48,49], and there were few studies on the low-temperature coating methods of solid electrolytes. It is worth noting that the content of the coating layer would have a significant impact on the specific capacity of the cathode material. Zhao et al. [50,51] studied the coating of Li_2_O-Al_2_O_3_-TiO_2_-P_2_O_5_ (LATP) on the surface of LNMO at a high temperature of 650 °C and suggested that an excessively thick solid electrolyte coating would inhibit the transfer process of electrons during charge and discharge [41].

In this study, the low-temperature treatment was adopted to form a hybrid coating of LATP/C on the surface of LNMO, which prevented the cathode material from being reduced by high temperatures and simultaneously improved the ionic and electronic conductivities. Specifically, a composite coating adhered with lithium iodide was reinforced by mechanical ball milling, and then a low-temperature processed LATP/C-LNMO core–shell structure was successfully constructed. The effect of the lithium iodide was to evoke a trace reduction in metal Mn^4+^ on the surface of the cathode electrode materials; thereafter, a higher ion diffusion rate of LATP/C-LNMO was achieved by the promotion derived from the enhancement of Mn^3+^ ions. Both the performance of LATP/C-LNMO and the original LNMO under high-voltage and high-temperature conditions were studied. The results confirmed that the electrochemical properties of LATP/C-LNMO with different LATP/C coating amounts were better than intrinsic LNMO, regardless of whether the reactions took place at room temperature or at high temperature.

## 2. Experimental Section

### 2.1. Preparation of LATP and LATP/C-LNMO Composite Materials

The Li_1.3_Al_0.3_Ti_1.7_(PO_4_)_3_ powder was synthesized via a sol–gel method which consisted of five main steps. Firstly, Ti(OC_4_H_9_)_4_ was added drop by drop to a solution of citrate acid under stirring conditions. Secondly, LiNO_3_, Al(NO_3_)_3_·9H_2_O, and NH_4_H_2_PO_4_ were added to the above hydrolysis solution with the stoichiometric amount of Li:Al:Ti:P by 1.3:0.3:1.7:3 M. Thirdly, an appropriate amount of ethylene glycol was added to promote gelation. Fourthly, the obtained gel was heated at 180 °C for 2 h and further sintered at 700 °C in the air for 4 h. Fifthly, the resulting product was refined using a mechanical ball mill and dried to obtain LATP powder [37]. LATP and porous carbon powder were mixed into the ethanol dissolved lithium iodide using a simple ball milling process, then the lithium iodide was recrystallized to serve as a binder when ethanol was vaporized at a low temperature to coat a uniform thickness and homogeneously distributed LATP/C composite on the surface of the LNMO cathode.

The LATP/C-coated LNMO composite materials were prepared using a two-step coating treatment process. Namely, LiI was coated on the surface of LNMO materials by evaporative crystallization and then LATP/C powders were embedded into the LiI layer via mechanical ball milling. The specific steps of this process are as follows. Initially, the lithium iodide powders (0.25 g) were uniformly dissolved into an ethyl alcohol solution. Then, the mass ratio amounts (LATP/LNMO = 2.5 wt.%) of pristine LNMO powders (10 g, Zhichuan technology, Co., Limited, Shenzhen, China) were added to the above-obtained solution under magnetic stirring to obtain the LiI-coated active materials (LiI-LNMO). Next, solid electrolyte power LATP (0.5 g), conductive carbon Super-P (0.5 g), and the above-mixed solution were transferred into a ball mill jar and ball milled for 2 h at a rotational speed of 240 rpm/min to embed LATP/C into the lithium iodide layer. Finally, the obtained slurry was heated at 80 °C for 0.5 h and then cured at 250 °C for 3 h before it was cooled down to room temperature to remove all solvents and achieve lithium iodide evaporation recrystallization. The final powder obtained was the LATP/C modified LNMO composite cathode material.

**Figure 1:**
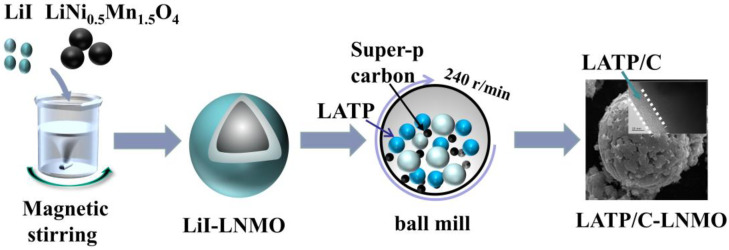
The schematic diagram of LATP/C coated LNMO composites materials.

Figure 1 shows the schematic diagram of the LATP/C coated LNMO composite materials. The resulting product was named 5% LATP/C-LNMO. For comparison, the LATP/C composite modified LNMO samples with 2.5 wt.% and 7 wt.% of LATP/C were treated with the same method, and the pristine LNMO was chosen as a control group without any additional modification for later characterization.

### 2.2. Materials Characterization

Powder X-ray diffraction (XRD) measurement with Cu Kα radiation was carried out to analyze the crystal structures of the coated materials. The range of diffraction data collection was 2*θ* = 10–70°. After refinement, the lattice parameter values of the material were obtained. The Raman spectra were characterized by a Raman spectrometer (LabRAM Odyssey). X-ray photoelectron (XPS) was applied to detect element valence on the surface of materials. The morphologies and structure of all obtained LATP/C coated and un-coated LNMO cathode materials were observed via a field scanning electronic microscope (SEM), which was used to obtain the element mapping pattern (EDS). The high-resolution TEM (HRTEM) images were collected on a JEOL-JEM 2100F instrument.

### 2.3. Electrochemical Characterization

Electrochemical performance tests were carried out in CR2032 coin cells with lithium metal negative electrodes. Next, 80 wt.% prepared active materials, 10 wt.% conductive carbon Super-P, and 10 wt.% polyvinylidenes fluoride (PVDF) were mixed in the N-methyl pyrrolidone (NMP) to prepare the cathode slurries. The prepared slurry was applied flat on the aluminum foil with a coating machine and then dried at 80 °C and 120 °C for 2 h, respectively. Finally, the coated Al foils were vacuum dried at 120 °C for 6 h before cutting into 14 mm discs. The mass loading of the active materials was about 3 mg cm^−2^. The CR2032 coin cells were all assembled in a glove box under an Ar atmosphere. The CR2032 coin cell consisted of the Celagard 2500 polypropylene separator (Celgard, LLC, Charlotte, NC, USA) and the electrolyte made of 1 M LiPF_6_ in the ethylene carbonate (EC)/dimethyl carbonate (DMC) (1:1 ratio by volume). All cells were measured using a CT2001A battery test system (Wuhan battery technology co., LTD, Wuhan, China) within a voltage of 3.5~5.0 V at various charge and discharge rates (0.1 C, 0.2 C, 0.5 C, 1 C, 5 C, 10 C). Electrochemical impedance spectra (EIS) were measured with an AC potential of 5 mV in a frequency range of 10 mHz~1 MHz, and the cyclic voltammograms (CV) were examined at a scan rate of 0.1 mV s^−1^ using a Corrtest workstation (Wuhan, China).

## 3. Results and Discussion

### 3.1. Crystal Structure and Surface Morphology of the Composite Cathodes

**Figure 2:**
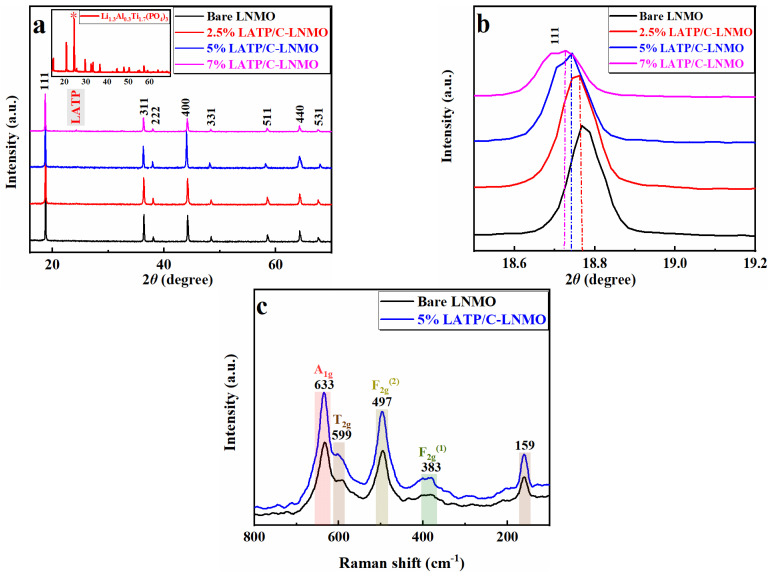
XRD patterns of (**a**) the bare LiNi_0.5_Mn_1.5_O_4_, LATP/C-LNMO and LATP powder and (*) the characteristic peaks. (**b**) enlarged view of the (111) diffraction peak (**c**) Raman spectra of the bare and 5% LATP-modified LNMO materials.

Powder X-ray diffraction was measured to identify the structure of samples and analyze the effect of the coating on the crystal structure of the active materials. Figure 2 displays the XRD patterns of LNMO samples before and after modification. There was no apparent difference between the diffraction peaks of LATP/C coated composites and the bare spinel LNMO. In detail, diffraction peaks of all samples were in accordance with the cubic *Fd3m* space group of the disordered spinel structure, where Ni^2+^ and Mn^4+^ underwent disorderly distribution at the 16a site of the octahedron, and the Li^+^ was at the 8a site of the tetrahedron. The relatively narrow peak shape indicated the excellent crystallinity of all composites. Furthermore, obvious diffraction peaks belonging to LATP were observed at 7% LATP/C-LNMO, which confirmed the existence of LATP in LATP- LNMO samples.

To quantify the phase structure changes of LNMO after LATP/C modification, the lattice parameters of all LNMO-based materials were calculated through Rietveld refinement of XRD; these parameters are listed in Table 1. It is worth noting that the lattice parameters (8.1714, 8.1885, 8.1891) of LATP/C-LNMO increased along with the amount of LATP in the coating layer, which was significantly higher than the bare LNMO (8.1666). This phenomenon was manifested by a slight shift in the peak position to the lower angle range in the XRD spectrum. One piece of circumstantial evidence was the degree of (111) diffraction peak shift from 18.767° to 18.728°, as shown in Figure 2b. The enlarged lattice parameters were attributed to the ion doping (Al^3+^ and Ti^4+^) and reduction in trace Mn^4+^ resulting from lithium iodide, which could be verified by a larger ionic radius of Mn^3+^ (0.645 Å), Al^3+^ (0.535 Å) and Ti^4+^ (0.610 Å) compared with Mn^4+^(0.530 Å) [45]. Thus, larger spacing of the lithium layer was confirmed by the increased lattice parameters after LATP/C modification, which would facilitate the diffusion of lithium ions in subsequent.

Figure 2c shows the Raman spectra of the bare LiNi_0.5_Mn_1.5_O_4_, LATP/C-LNMO. It can be seen that the LATP/C-modified material showed similar Raman spectra features to those displayed by bare LNMO. In detail, the peak of A_1g_ (633 cm^−1^) resulted from the symmetric Mn-O stretching vibration of MnO_6_ groups and the peaks located at 383 cm^−1^ and 497 cm^−1^ originated from the Ni^2+^-O stretching mode (F_2g_). The unsplit peak around 599 cm^−1^ was usually regarded as characteristic of the *Fd3m* disorder structure [52,53]. Moreover, the maxima half-width (FWHM) of A_1g_ and F_2g_^(2)^ in the LATP/C modified materials were higher than the bare LNMO, further confirming a disordered structure of LNMO, as revealed in the XRD results.

**Figure 3:**
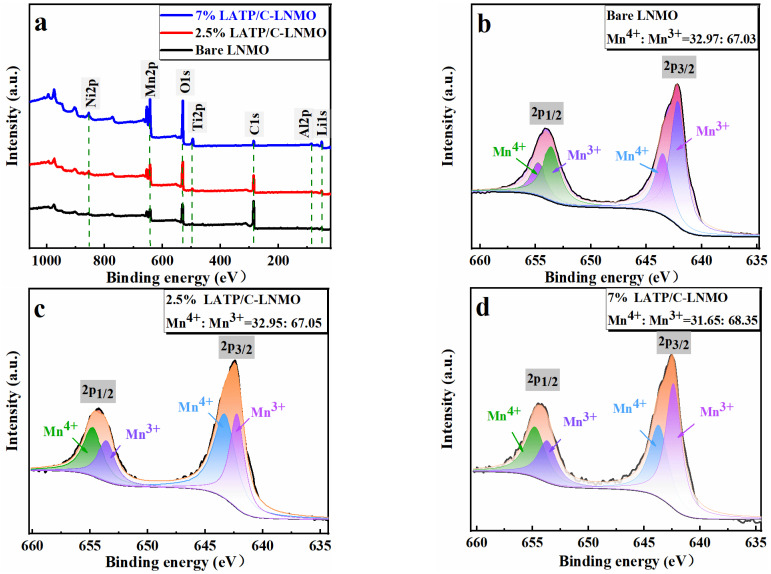

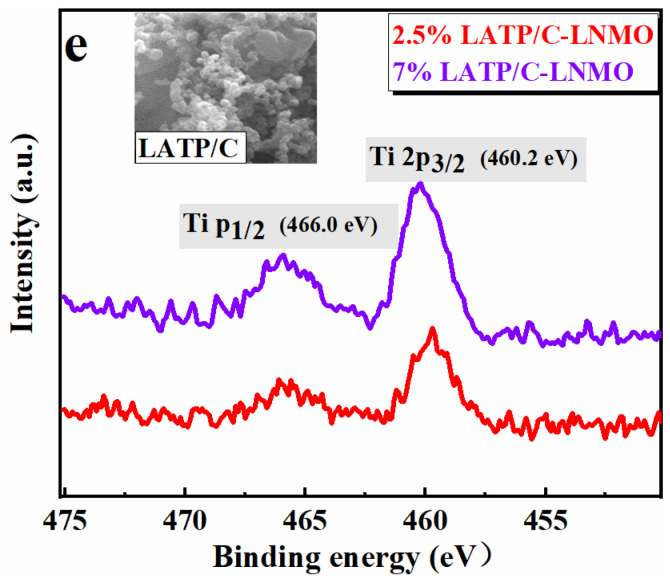
X-ray photoelectron spectroscopy collected at Mn2p and Ti2p regions of the bare LNMO and 2.5% LATP/C, 7% LATP/C coated composites and the SEM image of LATP/C.

X-ray photoelectron was performed for the analysis of sample surfaces; the results are displayed in Figure 3. It can be seen that, compared with the spectra of bare LNMO sample, elements of Al and Ti appeared on the LATP/C-modified materials, in which Ti (460.2 eV and 466.0 eV) corresponded to Ti2p_1/2_ and Ti2p_3/2_, respectively. The appearance of Al and Ti could also imply the LATP layer succeeded in covering the surface of LNMO. In the Mn2p_3/2_ spectrum, the peaks at 642.8 eV and 643.5 eV were assigned to Mn^3+^ and Mn^4+^, respectively. In Mn2p_1/2_, the electron peaks of Mn^3+^ and Mn^4+^ were 653.5 eV and 654.8 eV. It could be noted that the valence of Mn in LiNi_0.5_Mn_1.5_O_4_ and LATP/C-LNMO composites was between +3 and +4. To further confirm the reduction in Mn^4+^, the relative content of Mn^4+^ and Mn^3+^ was estimated by calculating the 2p_3/2_ peak area in all samples. The relative contents of Mn^4+^: Mn^3+^ in LNMO, 2.5% LATP/C-LNMO, and 7% LATP/C-LNMO were 32.97:67.03, 32.95:67.05 and 31.65:68.35, respectively, which was consistent with the XRD analysis. The lower the Mn^4+^ content, the higher the degree of cation disorder, which was favorable for Li^+^ diffusion and the electrochemical performance of LNMO cathodes in Lithium batteries.

**Figure 4:**
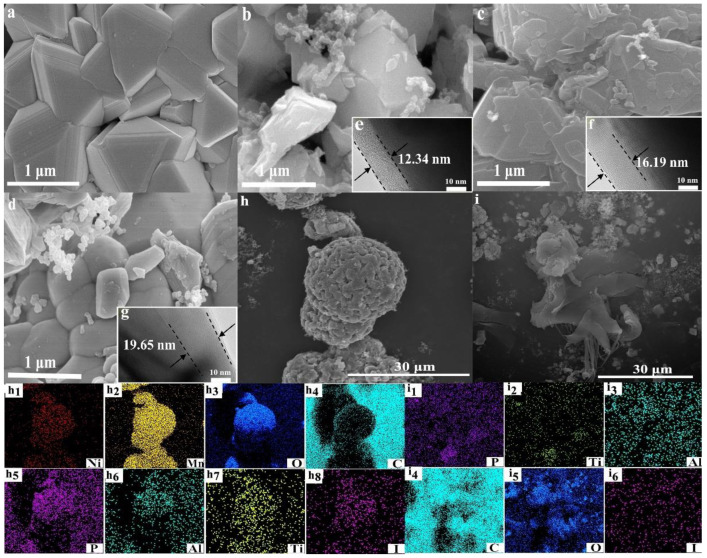
The SEM images (×40.0 K) of (**a**) the bare LNMO, (**b**) 2.5% LATP/C-LNMO, (**c**) 5% LATP/C-LNMO, (**d**) 7% LATP/C-LNMO, (**e**–**g**) the HRTEM images of LATP/C-LNMO**,** SEM with the corresponding EDS mapping images (**h_1_**–**h_8_** and **i_1_**–**i_6_**) for the area of (**h**) 5% LATP/C-LNMO and (**i**) LATP.

SEM analysis of different magnifications was carried out to observe the morphology and element distribution of the bare and LATP/C-coated LNMO. As shown in Figure 4b–d), apparent microspheres were observed on the surface of the coated composites compared with the smooth surfaces of the bare LNMO (Figure 4a), which illustrated that LATP/C adhered well to the surface of LNMO materials. Moreover, compared with bare LNMO particles (a typical octahedral shape), more contacted LATP/C- LNMO particles with round edges were observed with as the coating amounts grew. The rounded structure could be contributed by the coating of a LATP/C-doped lithium iodide film on the surface of the LNMO ball, and the protruded particles originated from the LATP/C complexes chimeric in the cladding layer.

Figure 4e–g display the HRTEM images of LNMO after modification. It can be seen that the thickness of the coating layer enhanced from 12.34 nm to 19.65 nm with the increase in the LATP/C content. Additionally, a reduced spacing between the active materials was presented as the filling effects of the LATP/C coating layer. Meanwhile, the tight-knit microspheres were meaningful, reducing the ion transfer resistance at the interface and mitigating the HF attack on the bulk structure. Thus, the LATP/C layers are verified to be well attached to the surface of LNMO.

The EDS mapping was conducted to illustrate the distributions of main elements on the spherical surface. Figure 4i,i_1_–i_6_ display the micromorphology and energy dispersive properties of the LATP/C, in which a homogeneous distribution of corresponding elements (i.e., Al, Ti, I, and C) was captured. This phenomenon implies that the strong bond between LATP and carbon would facilitate the formation of a film-like structure under the adhesion effects of lithium iodide. In particular, for the 5% LATP/C-LNMO (Figure 4h), a large number of nanospheres could be found on the surface of the LNMO microsphere. Correspondingly, as shown in EDS mapping images (Figure 4h_1_–h_8_), the homogeneous distribution of P, Ti, Al, and I elements on the surface of 5% LATP/C-LNMO indicated the adherence of the coated LATP/C layer resulting from the solidification of lithium iodide.

### 3.2. Electrochemical Properties

**Figure 5:**
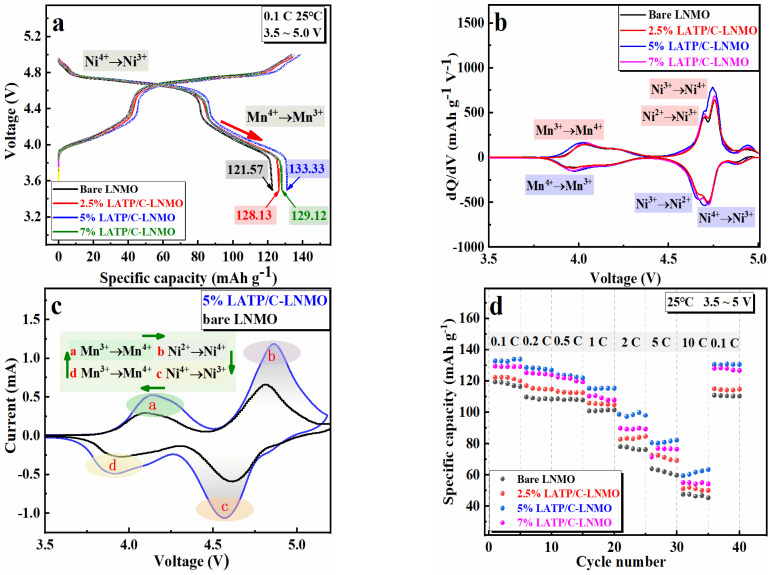
(**a**) Charge and discharge performance curves of the bare and LATP/C coated LNMO electrodes at 0.1 C, (**b**) the dQ/dV curves of the bare and LATP/C coated composite electrodes, (**c**) the CV curves of 5% LATP/C-LNMO and bare LNMO cathode at 0.1 mV s^−1^, (**d**) various rates (0.1 C, 0.2 C, 0.5 C, 1 C, 2 C, 5 C,10 C) discharge performance comparison of the bare and the LATP/C-coated composite electrodes.

Figure 5 illustrates the cathode of lithium batteries modified by the LATP coating. A series of galvanostatic current charge–discharge performance tests were implemented under different experimental conditions, which were subjected to half cells in a voltage range of 3.5~5.0 V. Figure 5a shows the initial discharge capacities for bare LNMO and LNMO coated with 2.5, 5, and 7 wt% of LATP/C were 121.57 mAh g^−1^, 128.13 mAh g^−1^, 133.33 mAh g^−1^, and 129.12 mAh g^−1^, with initial Coulombic efficiency values of 91.23%, 95.45%, 96.39%, 96.35%, respectively. The larger initial charge–discharge-specific capacity of the LATP/C coated LNMO electrodes indicated an improved electrochemical performance compared with the bare LNMO electrode. Additionally, both the charge–discharge curves of LATP/C coated LNMO and the bare LNMO electrode presented two typical platforms, in accordance with the CV results in Figure 5c. One was a long and smooth platform near 4.7V, caused by Ni^2+^/Ni^3+^ and Ni^3+^/Ni^4+^ redox reactions. Another was a short and inclined platform around 4.0 V, which resulted from the Mn^3+^/Mn^4+^ redox process. The discharge capacities of bare and LATP/C coated LNMO cathode between 3.5~4.3 V are 39.35 mAh g^−1^, 43.06 mAh g^−1^, 45.62 mAh g^−1^, and 43.53 mAh g^−1^, corresponding to the capacity ratio of 32.37%, 33.61%, 34.27%, and 33.72%, respectively, were attributed to the reduction in Mn^4+^/Mn^3+^. Namely, the enhanced retention rate of the coated composite electrode was probably ascribed to a greater amount of reduced Mn^4+^ ions. This phenomenon verified the enhancement of Mn^3+^ in LNMO after modification, which was consistent with the discussions in the XPS analysis.

Figure 5b shows the initial dQ/dV plot of all samples, which reflects the redox process and phase change reaction within the charge and discharge process. It can be seen that the peak at 4.7 V was split into two peaks, which were Ni^2+^/Ni^3+^ and Ni^3+^/Ni^4+^ redox couples, respectively. Meanwhile, the peaks at 4.0 V were mainly formed by the Mn^3+^/Mn^4+^ redox reaction. Furthermore, a tiny evolution was that the intensity of the Mn^3+^/Mn^4+^ redox peaks between 3.5 V and 4.2 V grew more prominent with the increase in the coating content. This phenomenon was also attributed to the increase in Mn^3+^ and the structure disorder, matching with the quantitative analysis of Mn^3+^ from XPS and charge–discharge curves.

Figure 5d exhibits the rate performance of the bare and LATP/C modified LNMO cathodes in 3.5~5.0 V. The half cells were discharged at different C-rates from 0.1 to 10 C, while the charge rate was fixed at 0.1 C. The discharge capacities of all samples tended to decrease with a higher discharge current, which was primarily influenced by polarization. Obviously, the LATP/C-LNMO cathode exhibited more excellent reversibility than the bare LNMO, especially at a low discharge rate from 0.1 C~2 C. It is noteworthy that 5% LATP/C-LNMO was accompanied by the smallest capacity fading, indicating the best reversibility among the coated cathode materials and a certain degree of mitigation of the polarization phenomenon on the battery. In particular, at a discharge rate of 2 C, a relatively significant capacity retention rate appeared in 5% LATP/C-LNMO compared with other modified cathodes. Specifically, the rated capacity was 106.07 mAh g^−1^ at 2 C for the 5 wt% coating amount of LATP/C and the contrasted values were 78.09 (bare), 82.7 (2.5%), 89.82 mAh g^−1^ (7%). In brief, the LATP/C-modified cathode materials presented a more stable enhanced performance, in which the LATP/C-LNMO with 5% coating content exhibited the best rate performance among the coated cathode materials.

### 3.3. Cyclic Behaviors and Kinetic Properties

**Figure 6:**
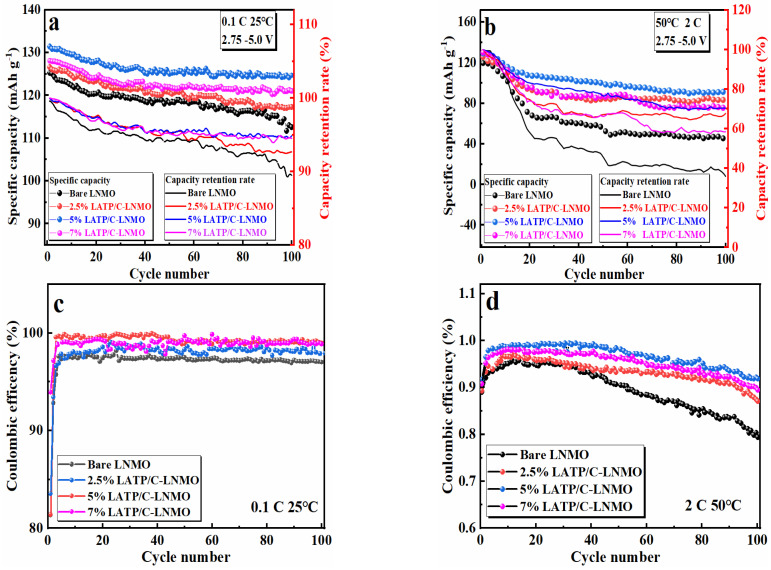

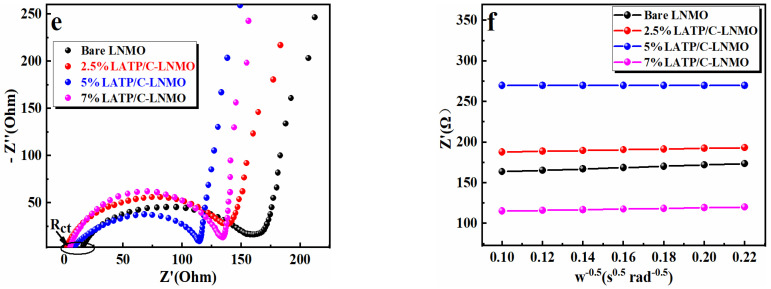
The cyclic performance of the bare and LATP/Ccoated LNMO composite electrode for 100 cycles: (**a**,**c**) at 0.1 C and room temperature; (**b**,**d**) at 2 C rate and 50 °C, the EIS plots (**e**) and Z’ vs. ω^−0.5^ plots (**f**) of pristine LNMO and LATP/C-LNMO.

Figure 6a shows the cycle stabilities and capacity retention rates of bare and LATP/C composite-coated LNMO cathode cathodes. The measuring conditions were a voltage ranging from 3.5 V to 5.0 V alongside a current density of 0.1 C over 100 cycles at room temperature. Notably, the discharge-specific capacity decreased to various degrees with the increasing number of cycles, which was blamed for the structural changes and side reactions of the cathode material. For the LATP/C-coated cathode materials (2.5%, 5% and 7% LATP/C-LNMO), the discharge capacities were 126.53 mAh g^−1^, 131.38 mAh g^−1^ and 128.15 mAh g^−1^ after 100 cycles, with corresponding capacity retention rates of 92.64%, 94.87% and 94.51%, respectively. In contrast, the cycling performance of the bare LNMO electrode was unsatisfactory; the capacity fading from 125.26 mAh g^−1^ decreased to 112.55 mAh g^−1^ after 100 cycles and the residual capacity retention rate was only 89.85%. Thus, the LATP/C-modified LNMO material tended to improve cycling performances and the 5% LATP/C was the most appropriate, demonstrating the highest capacity and the best capacity retention after 100 cycles in all samples.

Previous studies reported that severe side reactions between the cathode and the electrolyte would evoke rapid capacity fading at high temperatures [10,11], while the LATP/C was believed to inhibit the corrosion of liquid electrolytes on the metal (Mn) at high temperatures. Thus, a series of validation experiments were conducted, in which long-term stability tests for 100 cycles at 50 °C, with a discharge current of 2 C, and a detailed cyclic performance were displayed in Figure 6b. It can be seen that the capacity of the bare LNMO cathode demonstrated a striking decrease after 100 cycles, which might be caused by the decomposition of the electrolyte at high temperatures and the irreversible phase transition of the cathode structure. Compared with the bare LNMO, the magnitudes of enhanced capacities in LATP/Cmodified LNMO cathodes were 42.20 mAh g^−1^, 47.96 mAh g^−1^, and 33.13 mAh g^−1^, and corresponding capacity retention rates were improved to 66.78%, 70.40%, and 58.51%, respectively (35.96% for bare LNMO). Evidently, all LNMO materials coated with LATP/C composite exhibited a higher capacity retention rate than the bare LNMO, among which the 5% LATP/C-LNMO samples exhibited the best cycling stability performance. In addition, the LATP/C modified LNMO materials delivered a more stable coulombic efficiency than bare materials at 0.1 C and 2 C rates, as shown in Figure 6c,d. The reason for the improved cycling performance should be ascribed to the fact that the LATP/C layer coated on the surface of the active material slowed down the side reaction between the electrolyte and the cathode body within a long cyclic process, protecting the integrity of the cathode crystal structure.

To further study the influence of the LATP/C, half-cells batteries assembled with cathode materials with different amounts of LATP/C coating were tested to determine their electrochemical impedance between 0.1 Hz and 10^5^ Hz at various frequencies. Figure 6e shows the Nyquist diagram of the bare LNMO and the LATP/C-LNMO cathodes. The spectral lines of the test materials consisted of a semicircle and a straight line. It can be seen that the intersection of the semicircle in the Z’ axis was similar to the charge transfer resistance (R_ct_) at the cathode/electrolyte interface, and the R_ct_ of the LATP/C coated cathodes was significantly lower than that of the unmodified LNMO. The detailed values were listed in Table 2. The lower R_ct_ indicated that the coated cathode materials were more favorable to transferring lithium ions at the cathode/electrolyte interface, and the coating layer was inclined to reduce the interfacial resistance. The straight line corresponds to the ion diffusion process between the particles of the cathode material, which is known as the Warburg impedance. The lithium-ion diffusion coefficient can be calculated by the following formulas [39,54]:

(1) $$D_{Li^{+}}=\frac{R^{2}T^{2}}{2n^{2}A^{2}F^{4}C^{2}\sigma^{2}}$$

(2) $$Z^{\prime }=R_{s}+R_{ct}+\sigma w^{-1/2}$$

where *R* is the gas constant, *T* is the absolute room temperature (298.15 K), *R_ct_* is the interface charge transfer resistance, *R_s_* is the ohm resistance, *A* is the electrodes’ contact area, n is the electron transfer amount per mole (*n* = 1), *F* is Faraday’s constant, *C* is the Li^+^ concentration in the electrode and σ is the Warburg coefficient which can fit with the line w^−1/2^ by Z’ (Figure 6f). The slopes of the four samples were calculated by Equation (1) and the obtained D_Li+_ were 3.51 × 10^−12^ cm^2^ s^−1^, 4.90 × 10^−12^ cm^2^ s^−1^, 3.04 × 10^−11^ cm^2^ s^−1^ and 1.55 × 10^−11^ cm^2^ s^−1^, respectively. In comparison, the D_Li+_ values of the core cathode materials with the LATP/C coating exhibited excellent ionic conductivity that improved by one order of magnitude. In particular, the D_Li+_ of 5% LATP/C was confirmed to be the best among the coated cathodes, with results approximately eight times greater than that of the bare LNMO. All in all, the results mentioned above revealed that the LATP/C layer could not only decrease the electrode polarization but could also promote the fast Li^+^ transfer of the LNMO cathode during the ion insertion and extraction process.

## 4. Conclusions

In summary, a novel low-temperature heat treatment succeeded in synthesizing high-performance LATP/C-coated LNMO particles. The principle was to coat the LATP/C on the surface of the high-voltage cathode LNMO utilizing the lithium iodide with a solvent recrystallization property. A series of experiments with different mass percentages implied that a 5% LATP/C-LNMO material exhibited the best electrochemical properties. The optimized capacity of LNMO material with 5 wt% LATP/C reached 133.33 mAh g^−1^ at 0.1 C and the retention rate achieved 70.40% after 100 cycles at 2 C, which suggested the continuous LATP/C layer could effectively suppress interface side effects and stabilize the bulk structure. In addition, the 5% LATP/C-LNMO displayed a higher ionic conductivity of 3.04 × 10^−11^ cm^2^ s^−1^, which could be attributed to the trace Mn^4+^ to Mn^3+^ by I^−^ and the ion transport provided by the LATP on the surface of the active material. Thus, surface coating with lithium iodide was confirmed to be effective technology for cathode modification; it can improve the cathode/electrolyte interface and enhance the electrochemical performance.

## Figures and Tables

**Table 1:** Unit cell data of bare LNMO and LATP/C-LNMO.

	a	b	c	V(Å^3^)
LNMO	8.1666	8.1666	8.1666	544.67
2.5% LATP/C-LNMO	8.1714	8.1714	8.1714	545.62
5% LATP/C-LNMO	8.1885	8.1885	8.1885	549.05
7% LATP/C-LNMO	8.1891	8.1891	8.1891	549.17

**Table 2:** Impedance data of bare LNMO and surface modified LATP/C-LNMO samples in equilibrium.

	Bare LNMO	2.5% LATP/C-LNMO	5% LATP/C-LNMO	7% LATP/C-LNMO
R_s_ [Ω]	15.35	2.17	8.04	5.14
R_ct_ [Ω]	142.45	135.43	106.84	129.41
D_Li+_ [cm^2^ s^−1^]	3.51×10^−12^	4.90 × 10^−12^	3.04 × 10^−11^	1.55 × 10^−11^

## Data Availability

Data is contained within the article.
